# Recent challenges and advances in genetically-engineered cell therapy

**DOI:** 10.1007/s40005-017-0381-1

**Published:** 2017-12-28

**Authors:** Seok-Beom Yong, Jee Young Chung, Yoonsung Song, Yong-Hee Kim

**Affiliations:** 1 Department of Bioengineering, Hanyang University, Seoul, 04763 Republic of Korea; 2 Institute for Bioengineering and Biopharmaceutical Research, Hanyang University, Seoul, 04763 Republic of Korea; 3 BK 21 Plus Future Biopharmaceutical Human Resources Training and Research Team, Hanyang University, Seoul, 04763 Republic of Korea

**Keywords:** Gene engineering, Cell therapy, Gene-modified cell therapy, Immune cell therapy, Stem cell therapy

## Abstract

Cells naturally sense and actively response to their environment. Cell-therapy has long been studied and shown therapeutic effects in various diseases. However, several hurdles should be overcome to improve cell-based therapy. Gene delivery-mediated cellular modification has shown improvement of cell function by obstacle gene silencing and therapeutic gene expression. Especially, CRISPR/Cas9-mediated genome editing is a very promising method for gene modification. In this review, we describe the recent advances in genetic modification for cell therapy. Stem cells are still promising source of cell therapy due to their self-renewal character and differentiation potential. Immune cells regulate the inflammatory response and immunization, which inspired various cell therapy using immune-regulatory cells. Conclusively, we emphasize the need to develop gene-modification-based cell therapy as potent future treatment.

## Introduction

Cells can naturally sense and response to the environment by their functionality and living-cells have been challenged for disease therapy. Along with development of biological engineering, various attempts on genetical modification for improvement of cell therapy have been made. Long-historical viral vector-based gene delivery and non-viral vector-based gene transfection have been reported to facilitate cell modification. Although viral vector-based delivery systems have shown high transfection efficacy, they have serious problems including immune responses and insertional mutagenesis such as ectopic chromosomal integration followed by oncogenic reactions. Synthetic materials have been developed with successful results in gene therapy for various diseases. Though non-viral vectors showed less transfection efficiency compared to viral vectors, the safety issues with reduced pathogenicity and immunogenicity make non-viral vector more likely enter the clinical trials. Otherwise, recent technology of genome editing, especially CRISPR/Cas9 system enables complicated gene editing. In this review, we describe recent development of cell-therapies based on ‘genetically-modified’ cells. The contents were separated into two parts including genetically-engineered stem cells such as MSCs and HPSCs, and genetically-modified immune cells such as dendritic cells, macrophages and T cells. Finally, we emphasize the ‘needs’ of genetic engineering for realization of cell therapy for future medicine.

## Gene-modified stem cell therapy

**Fig. 1:**
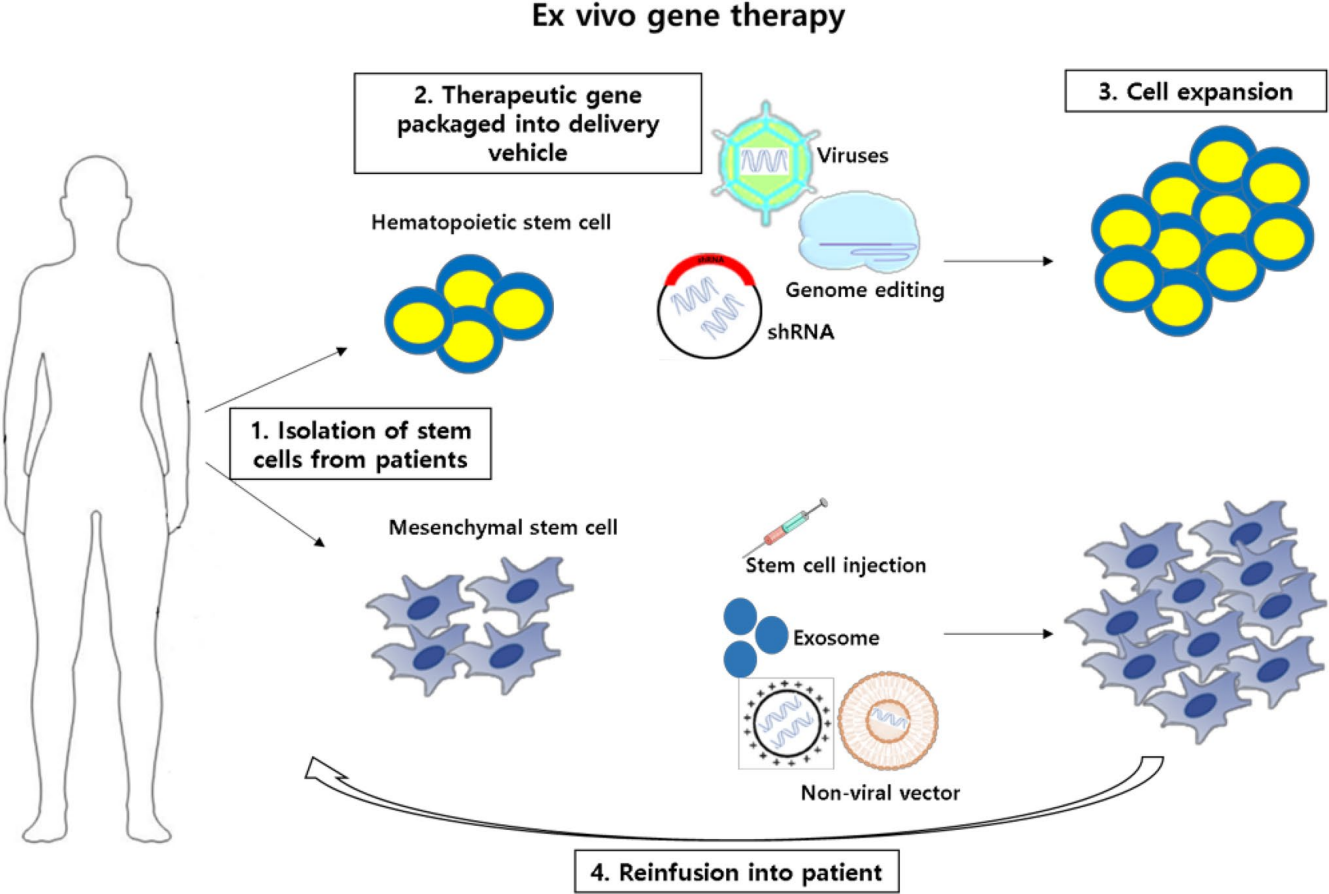
*Ex vivo* stem cell gene therapy

Stem cells have been gaining attention in regenerative medicine due to their self-renewal and multilineage differentiation. Cell therapy based on stem cells are widely used by introducing stem cells into tissues to treat diseases by gene therapy. Most stem cell therapies are based on hematopoietic stem cell and these tissue specific stem cells have now held a place in the cure for numerous diseases. Mesenchymal stem cells are also the most favored cell type in clinical studies due to its immunomodulatory properties. The clinical trials exploit improved vector systems to successfully delivery therapeutic genes. *Ex vivo* cell therapies has been of great interest as they are patients or normal donor oriented. Stem, progenitor or differentiated cells obtained from patients are expanded *ex vivo* by genetic modification and administered back into patients (Fig. 1).

### HSC gene therapy

Hematopoietic stem cell gene therapy (HSCs) have been the main target for *ex vivo* gene therapy thanks to the long clinical experience with HSC transplantation leading to highly integrated protocols (Naldini 2011). HSCs have the potential of self-maintaining multipotent to supply corrected gene progeny for hematopoietic lineage failure. The self-renewing nature of HSCs must be stably introduced into the cells either by vector mediated delivery or in situ gene editing (Thomas et al. 2003). The choice of gene delivery is dependent on the type of vectors used and integrating vectors derived from retroviruses [early generation vectors based on gamma-retroviruses (γ-RVs)] remain a preferred choice (Braun et al. 2014; Hacein-Bey-Abina et al. 2008; Stein et al. 2010). However, they also highlight the limitations and risks as they have limited ability to transfer genes and low transient expression of corrected hematopoietic cells in vivo. The development of lenti-viral vectors with improved efficacy and safety has been of great interest for *ex vivo* gene therapy. Several clinical trials using lenti-viral vector systems has been incorporated into severe inherited diseases of the immune systems such as Wiskott–Aldrich syndrome (WAS) (Abina et al. 2015; Aiuti et al. 2013) and X-linked severe combined immune-deficiency (SCID-X1) (Hacein-Bey-Abina et al. 2014) and β-thalassemia (Cavazzana-Calvo et al. 2010) and neurodegenerative disorders (Biffi et al. 2013; Cartier et al. 2009) (Table 1). Studies have focused on better vector development and better HSC processing methods but also focused on target gene editing to improve diseases such as disruption of the gene encoding a protein (Bcl11a) that repress expression of fetal globin for β-thalassemia (Wilber et al. 2010). Efforts have also been made for the treatment of β-thalassemia using siRNA by knocking down α-globin mRNA (Voon et al. 2008), zinc finger transcription factor Krüppel-like factor 1 (KLF1, also known as the erythroid Krüppel-like factor, EKLF) (Norton et al. 2017) or overexpression of its private chaperone, AHSP (Nasimuzzaman et al. 2010). As for the treatment of WAS caused by the mutation in the WAS gene, many retroviruses and lentiviruses that target the WAS gene have been researched on however the genome integration showed undesired off targets. Therefore CRISPR mediated genome editing targeting the WAS gene has been studied for treatment of the disease. SCID, an immune disorder characterized by absence of T and NK cells have been evaluated by correction of IL2RG gene (Schiroli et al. 2016).

**Table 1:** Stem cell gene therapy

Disease	Gene vector	Target cell	Target gene	Refs
HSC gene therapy WAS	Lentivirus CRISPR/Cas9	Multi lineage	Was specific gene	Aiuti et al. (2013); Boztug et al. (2010) Wang et al. (2015)
X-linked SCID	γ retrovirus	Lymphocyte Myeloid	IL2RG	Hacein-Bey-Abina et al. (2014)
β Thalassaemia	Lentivirus Lentivirus of shRNA	Erythrocyte	Bcl11a HMGA2 KLF1	Roosjen et al. (2014); Sankaran et al. (2008) Zhao et al. (2017) Amaya et al. (2013); Borg et al. (2012)
ALD	Lentivirus	Tissue macrophage and microglia	ABCD1	Cartier et al. (2009)
MLD	Lentivirus	Tissue macrophage and microglia	ARSA	Biffi et al. (2013)
MSC gene therapy GVHD	Stem cell injection Non-viral	MSC, monocyte T-cell	TGFβ CCR4, CCR8 Let7a	Lim et al. (2017) Yu et al. (2017)
Myocardial infarction	Exosomes	Cardiac muscle cell	Nrf2	Mu et al. (2014)
Ischemia	Non-viral	MSC	miR-133b	Huang et al. (2017)
Bone and cartilage repain	Biomaterial	BMSCs	BMP-2	Balmayor et al. (2017)

*HSC* hematopoietic stem cell, *MSC* mesenchymal stem cell, *WAS* Wiskott–Alrdich syndrome, *X-linked SCID* X-linked severe combined immunodeficiency, *ALD* adrenoleukodystrophy, *MLD* metachromatic leukodystrophy, *BMSC* bone marrow mesenchymal stem cell

HSCs gene therapy is administered by *ex vivo* gene transfer into hematopoietic progenitors by purifying CD34 surface marker from leukocytes obtained from bone marrow or peripheral blood. Then CD34 purified cells are cultured for approximately 4 days in presence of growth factors and vectors carrying an expression cassette for the corrective gene (Huang et al. 2016). Before administration of the modified cells, patient’s progenitor and differentiated cells are depleted in the bone marrow following chemotherapy. The depletion favors the engraftment of *ex vivo* corrected gene therapy; however, they also cause secondary tumors and infertility (Copelan 2006). To overcome these problems, several HSCs gene therapy have concentrated by lowering the chemotherapy dosage that are used for HSC transplantation, but they are yet to be determined. Lenti-viral HSCs gene therapy shows high level of hematopoiesis with the corrected genes in most patients and has been no report of adverse events related to lenti-viral gene therapy. Apart from lenti-viral therapy, genome editing by use of CRISPR system shows a great promise for hematopoietic stem cell therapy as more researchers are focusing on improved gene delivery system with efficient gene targeting (Mandal et al. 2014).

### MSC gene therapy

Mesenchymal stem cell (MSC) gene therapy have been widely used in clinical trials due to their heterogenetic properties (Bianco 2014). MSC are classified as postnatal, self-renewing multipotent stem cells that are capable of multilineage differentiation. They are also defined by their spindle shaped morphology, adhering capability in vitro and unique cluster expression during cell differentiation (Wei et al. 2013). Stromal MSCs are defined differently from classic MSC as they are classified from various tissues and shows the properties of fibroblastic markers and when transplanted modulate the host immune system. Due to their safety regarding the short existence, anti-inflammatory properties and homing to damage sites they are widely used for allogenic cell transplantations and clinical studies (Wang et al. 2016). For the past few years, the number of registered clinical trials of MSC gene therapy has increased but the distribution of clinical pipeline is still a major hurdle to overcome. The pleiotropic properties of MSCs provides a broad range for their potential in regeneration of organ tissues, immune related disorders and neurodegenerative diseases (Table 1) and moreover translational studies have proved their attractiveness for clinical use (Glenn and Whartenby 2014). By the end of 2016, there were over 500 clinical studies related to MSC therapy, the main clinical indications being autoimmune diseases and bone cartilage (http://clinicaltrials.gov/).

Immune suppression properties of MSC have important roles in suppressing activated T-cells and their host disease (GVHD), to overcome the serious consequence of GVHD MSC have been used in clinical studies due to their immunosuppressive properties. Several phase II clinical studies have shown lower transplant related mortality and higher survival rates when treated together with MSCs. Children suffering from stage III–IV GVHD received MSC and about 42% of the children survived for a medium of 611 days leading to approval of Prochymal MSC for severe pediatric GVHD in Canada and New Zealand. Apart from clinical studies, diverse researches has been carried out using gene targeting such as down-regulation of TGF-beta expression, inhibition of infiltration of immune cells via down-regulation of CCR4 and CCR8 on monocytes (Lim et al. 2017), microRNA based strategy by knock-down of let-7a to improve MSC immunotherapy (Yu et al. 2017). Apart from immunosuppressive properties, MSCs have been widely used for myocardial infarction, ischemia stroke, osteoarthritis and liver diseases (Buzhor et al. 2014). In myocardial infarction, allogeneic MSC transplants have shown improved ventricular ejection fraction and also the use of exosomes derived from MSCs have been widely used (Gonzalez-King et al. 2017). Various gene therapies have been researched on such as the peptide modified MSC using miR-133b for treatment of cerebral ischemia (Huang et al. 2017) also micelles were used for siRNA transfection into mesenchymal stem cells (Raisin et al. 2017). Bone marrow derived MSCs are widely used for bone and cartilage repair and in osteoarthritic patient’s intra-articular injection of MSC resulted in strong improvement of cartilage coverage (Jo et al. 2014) and modified mRNA for BMP-2 to induce osteogenic pathways in MSCs (Balmayor et al. 2017). Due to the advantage of the mesenchymal stem cells, stem cell therapy can be broadened to diverse diseases.

## Gene-modified immune cell therapy

**Fig. 2:**
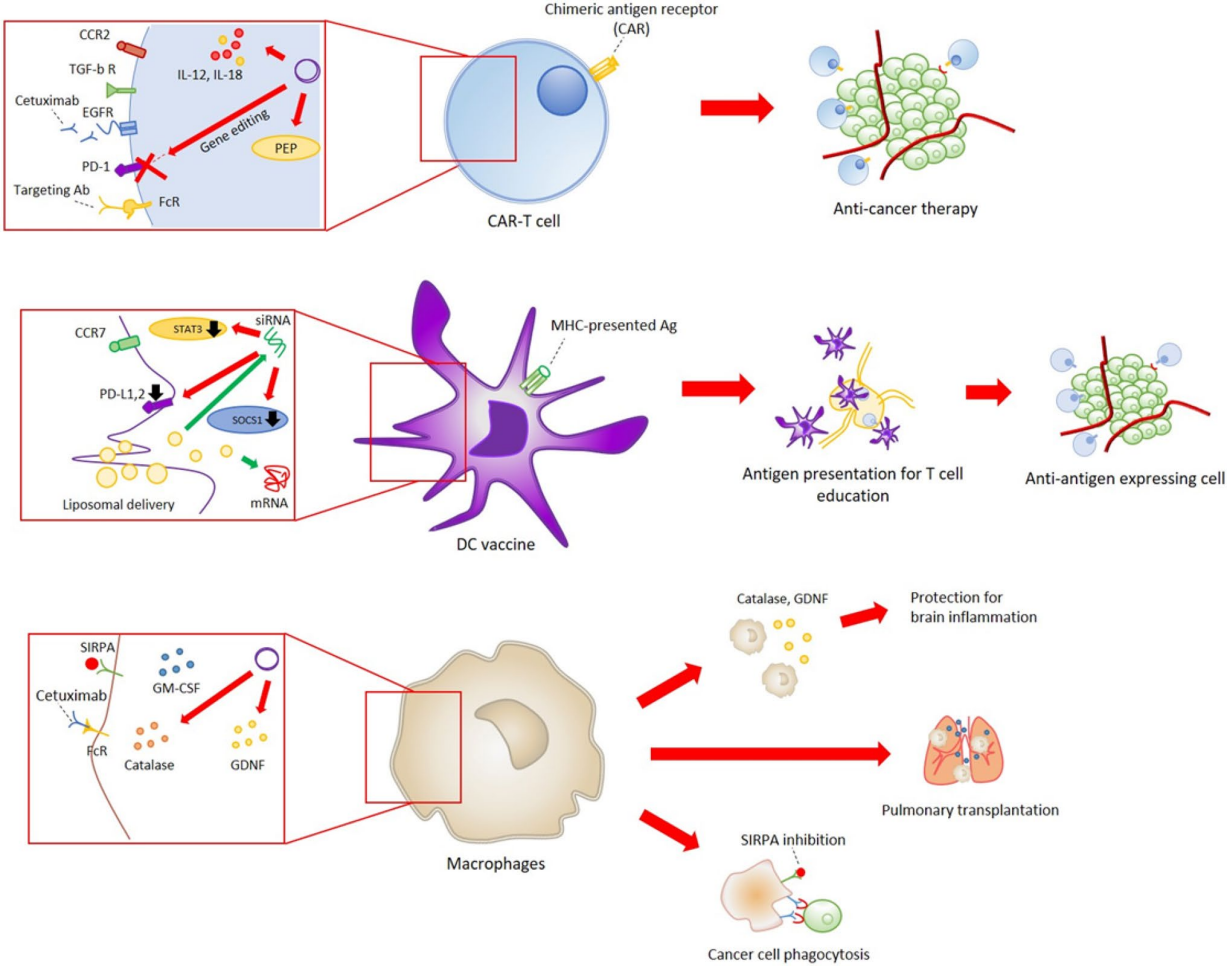
Gene-modified immune cell therapy

Immune cells are related to various disease pathologies such as inflammatory diseases, cancer, transplantation rejection (Grivennikov et al. 2010; Ross 1999). Due to major role of immune regulation, there are many types of gene-modified immune cell therapy with two main stream of dendritic cell vaccine and CAR-T cell for cancer therapy (Fig. 2).

### Gene modified-dendritic cell (DC) for improved tumor vaccination

DCs are professional antigen presenting cells which presents the phagocytosed and processed antigens to T cells via MHC and co-stimulatory factors (CD80, CD86). Antigen-presented naïve T cells expand and matured to CD4+, CD8+ effector T cells to attack, re-act to antigenic cells, especially for cancer. Due to its major role of antigen-presentation, DCs as natural adjuvant, DC-based cancer antigen vaccination has been studied for decades (Lesterhuis et al. 2011; Murphy et al. 1996; Nestle et al. 1998; Romano et al. 2011; Timmerman et al. 2002; Yu et al. 2001). Although with its antigen presenting and clonal T cell stimulating character, there have been hurdles of DC-vaccination; (1) enhancement of antigenic immunity and (2) breaking of immunosuppression by tumor microenvironment; and combinatory gene delivery for improvement of DC-tumor vaccination have been studied. Based on the immunosuppression mechanism in the dendritic cells by SOCS1 gene, lentiviral SOCS1 siRNA delivery reduced immunosuppressive effect in DC and improved antigenic response for T cell education (Shen et al. 2004). In consistency with SOCS1, siRNA targeting A20, the ‘attenuator of antigen presentation’ enhanced co-stimulatory factor such as CD80, CD86 and cytokine expression in DCs which overcomes regulatory T-cell-mediated immune suppression (Song et al. 2008). With discovery of PD-L1 and PD-1 interaction (Freeman et al. 2000) in tumor immune suppression, Hobo et al. delivered PD-L1, L2 ligand-targeting siRNA via electroporation to DCs. PD-L1,2 knock down-DCs shows reduced interaction with PD-1 on patient-derived T cells and enhanced antigen-specific T cell stimulation with proliferation, consequently boost DC-mediated vaccine effects (Hobo et al. 2010). With liposomal system, Akita et al. delivered siRNA for SOCS1 and improved the vaccinating effect of DCs for tumor vaccination in mice. R8-GALA peptide modified liposome shows better endosomal escape and gene knock down efficiency (Akita et al. 2010) and YSK12-C4 based YSK12-MEND shows dramatic gene delivery efficiency (~ 1.5 nM Km) which facilitates DC-gene silencing for lymphoma vaccination compared (Warashina et al. 2016). Spermine-dextran, a kind of cationic polymer-mediated gp100-melanoma antigen delivery showed anti-melanoma effect and CCR7 co-expression improved lymph node migration of transfected dendritic cells in vivo (Chen et al. 2013). Other than that, STAT3 siRNA and R837; immune modifier; containing PLGA nanoparticle improved the maturation and antigen presentation ability of DCs for OVA-cancer therapy (Heo and Lim 2014). Recently direct antigen expressing mRNA delivery also programmed DCs to present antigens for T cells. Liposome-mediated antigenic mRNA delivery processed MHC II-antigen presentation and induced cancer vaccination effect (Kranz et al. 2016). DC biology have shown heterogeneity in tissue and organ (Geissmann et al. 2010). Recent studies shows different antigen presenting capacity between tissue resident DCs and monocyte-derived DCs in muscle (Langlet et al. 2012) and tumor (Laoui et al. 2016) which suggest needs-for study on different function, optimization of type-dependent dendritic cell vaccination for future DC vaccine-mediated cancer therapy (Table 2).

**Table 2:** Gene-modified DC vaccines

	Gene vector	Target gene	Target gene function	Refs
DC vaccination	Lentivirus Lentivirus	SOCS1 A20	Immune suppressor Attenuator of antigen presentation	Shen et al. (2004) Song et al. (2008)
	R8-GALA-Lipo YSK12-MEND-Lipo Liposome	SOCS1 SOCS1 Antigenic mRNA	Immune suppressor Antigen expression	Akita et al. (2010); Warashina et al. (2016); Geissmann et al. (2010)
	Electroporation	PD-L1,2	Immune suppression	Roosjen et al. (2014); Sankaran et al. (2008)
	Spermine-dextran	CCR7	Lymph organ homing	Chen et al. (2013)
	PLGA nanoparticle	STAT3	DC maturation	Biffi et al. (2013)

### Gene modification for improved chimeric antigen receptor T cells (CAR-T cells)

Other than DC vaccines, are proper for antigen-specific effector T cell expansion and long term tumor immunizations, negative selection in T cell development against endogenous antigen harden the efficient therapy of self-antigen based DC vaccine (Palucka and Banchereau 2012). Based on the interactions of T cell’s CD3, C28 receptors with DC’s peptide-loaded MHC II, chimeric antigen receptor (CAR) expression-mediated T cell modification have shown efficient tumor regressions (Kershaw et al. 2006, 2013; Lamers et al. 2006; Park et al. 2007). Mostly well-known CAR-T cell is CD19-targeted one which recognize the CD19 on B cell lymphoma and destroyed them successfully in clinical trial (Brentjens et al. 2013; Davila et al. 2014; Lee et al. 2015). Other than CD19-targeted one, CAR-T cells on chronic leukemias and Her2-solid tumors have been studied (Ahmed et al. 2015; Kalos et al. 2011; Lynn et al. 2015; O’Hear et al. 2015). However, (1) tumor microenvironment-immune suppression and (2) T cell exhaustion, (3) targeting problem requires additional genetic modifications for CAR-T cell improvement. To overcome PD-1 and PD-L1 immunosuppression, extracellular domain of PD-1 was fused to intracellular costimulatory domain which activated T cells (Prosser et al. 2012; Ren et al. 2017; Schumann et al. 2015) removed PD-1 receptor from T cell genomic DNA by CRISPR/Cas9 system. The expression of dominant negative receptor form of TGF-beta enhanced immune activation state of T cells (Foster et al. 2008). Based on glucose-metabolic competition between cancer cell and T cell, genetically engineered T cells with increased phosphoenolpyruvate (PEP) expression showed enhanced effector function (Ho et al. 2015). Pro-inflammatory cytokine, IL-12, IL-18 expressing, ‘Armed’ T cells shows higher anti-tumor effect by stimulating both adaptive and innate immune systems (Boice et al. 2016; Zhang et al. 2015). To eliminate and control transferred-T cell efficiently, extracellular domain of EGFR was expressed on CAR-T cells responsive them to EGFR-antibody such as cetuximab (Wang et al. 2011). Adapter-CAR T cells are advanced form of targeting such as Fc receptor-expression for antibody-decoration (Kudo et al. 2014) and specific ligand binding domain-modification for ligand binding (Ma et al. 2016). With chemotactic migration of CCR2, additional CCR2 expression enhanced tumor accumulation and anti-tumor effect (Moon et al. 2011). Conclusively CAR-T cell modification is still unmet need with great potential for sophisticated cancer therapy (Table 3).

**Table 3:** Gene-modified CAR-T cells

	Target gene	Target gene function	Refs
CAR T cell	PD-1	Immune suppressor	Schumann et al. (2015) Ren et al. (2017)
	Negative form of TGF-beta receptor	Immune suppressor	Foster et al. (2008)
	PEP	Glucose metabolism	Ho et al. (2015)
	IL-12 IL-18	Immune activation	Boice et al. (2016); Zhang et al. (2015)
	EGFR	Cetuximab-binding for apoptotic induction	Wang et al. (2011)
	Fc receptor	Targeting antibody binding	Kudo et al. (2014)
	Ligand binding domain	Targeting ligand binding	Ma et al. (2016)
	CCR2	Chemotactic migration for tumor targeting	Moon et al. (2011)

### Gene-modified and armed macrophages

With disease-homing and accumulation character, macrophages are professional phagocytes and immune effectors (Geissmann et al. 2010). As previously described, macrophage accumulation and polarization induce the inflammatory state of disease area such as myocardiac infarction (Swirski et al. 2009) and atherosclerosis (Robbins et al. 2013), adipose tissue (Amano et al. 2014) and tumor (Franklin et al. 2014). Tumor educated-macrophages (tumor associated macrophage; TAM) polarized to M2-like macrophages suppressing inflammatory response and T cell activations for tumor raise and TAM-depletion mediated anti-cancers are very important issues (Mantovani et al. 2002). Due to effector functions, macrophages have been applied for human disease therapy in clinical trials, mostly shows no therapeutic effects (Knoller et al. 2005; Lammertse et al. 2012; Lee et al. 2016). For improved macrophage-therapy and its clinical success, there have been researches of gene-modified macrophage based therapy. For hereditary pulmonary alveolar proteinosis (herPAP), a disease induced by GM-CSF receptor mutation, Happle et al. (2014) and Suzuki et al. (2014) transplanted pulmonary with GM-CSF expressing macrophage (wild type). The transplanted macrophages differentiated to functional alveolar macrophages reduces proteinosis and enhanced the lung function for lasting 9 months. As previously mentioned tumor infiltrating monocytes and macrophages are a potential target for tumor therapy and Giulia Escobar et al. modified hematopoietic cells to express interferon alpha since their differentiation by using TIE2 promoter based lentiviral vector. Due to tumor infiltrating characteristic of TIE2-expressing monocytes, enhanced interferon-alpha response in tumor and reduced tumor growth was observed which suggest new-tumor immunotherapy (Escobar et al. 2014). With liposomal systems, Haney et al. (2011, 2013) and Zhao et al. (2014) delivered catalase and GDNF gene-expressing pDNA into macrophages and injected them intravenously. The macrophages were accumulated in brain and extracellular vesicles released from delivered-macrophages were delivered to neurons by protruding microtubules which reduces inflammation and neuronal destructions, consequently propose the therapy for Parkinson’s disease. Other than gene-transfection, antibody and drug-loaded nanoparticle modified macrophages are also promising topic. Anselmo et al. (2015) and Klyachko et al. (2017) engineered the Raw264.7 cells with non-phagocytic microparticle, ‘polymeric backpacks’ for anti-inflammatory drug delivery in brain and inflamed lung, skin. Recent paper of macrophage therapy introduces the antibody-blocking of SIRP-alpha inhibited CD47 interaction of macrophages on cancer cells which improves phagocytic uptake and removal of cancer. With cetuximab- and anti-SIRP-alpha-antibody modification of macrophages suppresses EGFR-lung tumor growth in mice (Alvey et al. 2017) (Table 4).

**Table 4:** Gene-modified and armed macrophages

	Gene vector	Target gene	Target gene function	Refs
Macrophage therapy	–	GM-CSF	Macrophage differentiation	Happle et al. (2014) Suzuki et al. (2014)
	TIE2-lentivirus	Interferon alpha	Anti-tumor immune response	Escobar et al. (2014)
		Catalase GDNF	Anti-inflammation, neuronal protection	Haney et al. (2011, 2013); Zhao et al. (2014)
		SIRPA	Phagocytic signaling	Alvey et al. (2017)

## Conclusion

Due to the natural character of the cells, they have been applied for therapy, genetic modification and engineering. They also have a potential for improvement of cell functions. Temporal gene silencing and expression by siRNA and pDNA has been the most valuable approaches and CRISPR/Cas9-mediated gene editing is a promising tool for future cell-engineering.
